# Spontaneous Preterm Births in Malaysia: Are There Modifiable Antenatal Risk Factors?

**DOI:** 10.7759/cureus.59152

**Published:** 2024-04-27

**Authors:** Narisa H Damanhuri, Noran N Hairi, Maslinor Ismail, Ravichandran Jeganathan, Shamala D Karalasingam, Muhammad Jaffri Mohd Nasir, Shahrul Aiman Soelar, Kamarul Imran Musa, Tengku Alina Tengku Ismail

**Affiliations:** 1 Department of Social and Preventive Medicine, Faculty of Medicine, Universiti Malaya, Kuala Lumpur, MYS; 2 National Obstetrics Registry, Hospital Sultanah Aminah, Johor, MYS; 3 Department of Obstetrics and Gynaecology, University of Cyberjaya, Selangor, MYS; 4 Faculty of Entrepreneurship and Business, Universiti Malaysia Kelantan, Kelantan, MYS; 5 Clinical Research Centre, Hospital Sultanah Bahiyah, Kedah, MYS; 6 Department of Community Medicine, School of Medical Sciences, Universiti Sains Malaysia, Kelantan, MYS

**Keywords:** malaysia, modifiable risk factors, antenatal factors, healthcare government facilities, spontaneous preterm birth

## Abstract

Background

Spontaneous preterm birth (SPB) is a global public health concern with devastating health effects on SPB survivors. This study aimed to determine modifiable antenatal risk factors associated with SPB among women attending government healthcare facilities in Malaysia.

Methodology

A retrospective record review of 49,416 national obstetrics registry data from 2015 was conducted and analyzed using binary logistic regression based on six antenatal factor divisions.

Results

Mothers with pre-existing diabetes had higher odds (adjusted odds ratio (aOR) = 3.09) of delivering prematurely than mothers without diabetes. Mothers with chronic hypertension with superimposed pre-eclampsia (aOR = 2.51) and gestational hypertension (aOR = 1.44) had higher odds of experiencing preterm birth than mothers with no hypertension. Underweight mothers had higher odds (aOR = 1.27) of delivering prematurely than mothers with an ideal body mass index (18.5 to <25.0 kg/m^2^). Mothers with moderate anemia (hemoglobin level: 7 to <9 g/dL) had higher odds (aOR = 1.18) of preterm birth than mothers with normal hemoglobin levels (≥11 g/dL).

Conclusions

Maternal biomarkers, such as glucose level, blood pressure, BMI, and hemoglobin level, play an important role in reducing the rate of SPB in Malaysia. This study recommends strengthening pre-pregnancy, antenatal, and postpartum care through multidisciplinary and multi-agency team collaboration, addressing both modifiable and non-modifiable risk factors and adopting a dual approach that combines preventive and curative care.

## Introduction

Preterm birth is the delivery of a live fetus before 37 completed weeks of gestation which can be classified as spontaneous or provider-initiated type. Spontaneous preterm birth (SPB) occurs due to preterm delivery preceded by either spontaneous preterm labor (with intact membranes) or preterm premature rupture of membranes. The provider-initiated type refers to preterm birth following medical interventions such as induction of labor or cesarean section due to maternal/fetal indications [1].

In 2010, an estimated 14.9 million preterm births occurred globally, accounting for approximately 11.1% of all live births, with an uncertainty range of 12.3-18.1 million [2]. In Malaysia, the preterm birth rate in 2010 was below 10.0% of the global burden of preterm birth [3,4]. However, this rate increased by 39.5% between 2010 and 2012 (11.3%, N = 124,096) [5]. Interestingly, this soaring rate plummeted from 22.1% (N = 122,096) in 2013 to 12.4% (N = 151,268) in 2015 [6]. Although these findings showed a marked downward trend in the proportion of preterm births in Malaysia, it is still above the latest global prevalence of 10.6% estimated in 2014 (N = 139.95 million with an uncertainty interval of 9.0-12.0 million) [7].

Despite the overall decline in preterm birth rates, preterm infants continue to experience serious health consequences. The immediate effects can be seen from the worrying mortality rates, i.e., deaths among the perinatal, neonatal, and infant age groups which are caused by complications of prematurity. Approximately three-quarters of perinatal deaths occur among preterm infants [8]. Out of four million neonatal deaths, roughly one-third were caused by preterm birth [2]. In addition, preterm infants contribute to the second leading cause of death among infants after pneumonia [9,10].

Morbidities associated with SPB are debilitating giving rise to a significant socioeconomic burden. This is evident from a study that demonstrated that across all ages, causes, and complications, SPB constitutes the eighth most common cause of disability-adjusted life years in developing countries [11]. Complications of SPB can be observed as early as during the immediate postpartum period involving many important body systems, including the immune, hematologic, gastrointestinal, central nervous, cardiovascular, and respiratory systems [12].

The long-term health effects of prematurity, often classified as irreversible consequences, can be broadly described based on three major domains which include sensory (hearing and vision), physical (motor development), and mental (neuro-development) [13]. These effects can be seen among preterm survivors who become afflicted with developmental, behavioral, and cognitive disorders such as attention-deficit hyperactivity disorder, autistic spectrum disorder, and other learning and social communication challenges. Moreover, preterm infants have a higher risk of permanent lifelong debilitating health problems, including metabolic syndromes, cerebral palsy, blindness, deafness, and developmental and behavioral abnormalities, which collectively increase the socioeconomic burden [13].

Most research has focused on understanding the pathophysiology of SPB from the point of uterine quiescence to active contractions leading to birth before 37 completed weeks of gestation [14]. Consistent research findings have shown that SPB is multi-factorial in origin, resulting from multiple interactions of maternal, fetal, environmental, and social factors [15,16]. Although the exact cause of SPB is still unknown, several evidence-based factors, both modifiable and non-modifiable, have been identified [17,18]. These factors can be broadly classified into the following six categories: environmental toxicants; genetics and constitutional; medical and pregnancy conditions; mental health, psychosocial, and behavioral; nutritional; and sociodemographic and community [17].

In this study, the term modifiable antenatal factors refers to factors (before and during pregnancy) that can be addressed, intervened, or managed to reduce the impact on the outcome of SPB. On the other hand, non-modifiable antenatal factors refer to factors (before and during pregnancy) that are inherent or in-built.

This study aims to determine the modifiable antenatal risk factors associated with SPB among women attending government healthcare facilities in Malaysia.

## Materials and methods

**Figure 1:**
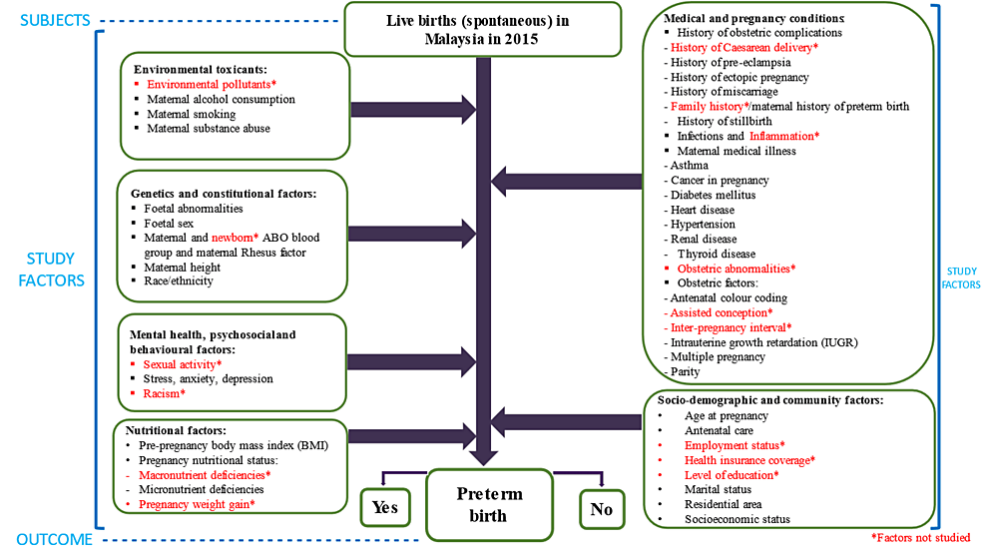
Conceptual framework. The asterisked study factors highlighted in red are excluded as they are unlisted under the National Obstetrics Registry’s database (study data source). Image credit: Narisa H. Damanhuri.

The conceptual framework of the study encompassing subjects, study factors, and outcomes is presented in Figure 1. Subjects refer to the spontaneous live births that occurred in Malaysia in 2015, study factors are the antenatal factors under study which were grouped under the six evidence-based antenatal factor divisions, and study outcome denotes whether the live birth was preterm or term birth. The conceptual framework of this study was drawn based on previous investigations on the six evidence-based antenatal factors of SPB [17] and the accepted theory by Shonkoff on the effect of early stimuli (antenatal factor) on pregnancy outcome [18].

The independent variables (explanatory variables) were divided into the following six antenatal factor divisions and their study factors: (1) environmental toxicants (maternal alcohol consumption, maternal smoking, and maternal substance abuse); (2) genetics and constitutional factors (fetal abnormalities, fetal sex, maternal ABO blood group and maternal Rhesus factor, maternal height, and ethnicity); (3) medical and pregnancy conditions (history of obstetric complications, infections, maternal medical illness, and obstetric factors); (4) mental health and psychosocial factors (psychiatric disorders including stress, anxiety and depression); (5) nutritional factors including pre-pregnancy body mass index (BMI at booking) and micronutrient deficiency (anemia at booking); (6) sociodemographic factors (age at pregnancy, antenatal care, marital status, residential area and socioeconomic status). The dependent variable (response variable) was SPB which was coded 1 for preterm birth and 0 for term birth at the statistical analysis stage.

Study design

**Figure 2:**
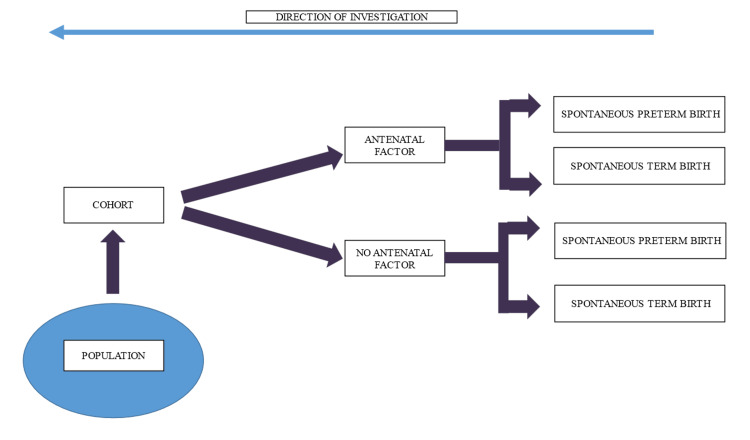
Graphic representation of the study design. This study examined a cohort of women whose outcome (spontaneous preterm birth versus spontaneous term birth) occurred in 2015 with the exposure (antenatal factor) occurring before the outcome. Data on exposure to antenatal factors (antenatal records) and data on prematurity and neonatal outcome (obstetric records) were reviewed in a retrospective manner (direction of investigation) to determine the associated antenatal factors of spontaneous preterm birth among this cohort of women. Image credit: Narisa H. Damanhuri.

A retrospective record review of 49,416 National Obstetrics Registry (NOR) data was conducted. Data on all births of women attending government healthcare facilities in Malaysia in 2015 was downloaded from the NOR’s database (N = 151,268). The data contained both antenatal and obstetric records of these women. A graphic representation of the study design is illustrated in Figure 2.

Study data source

The study data were sourced from the NOR. In 2015, this database collated 151,268 records of deliveries from its nationwide designated data site providers (DSPs) which comprised 13 tertiary state hospitals and one district hospital. These DSPs have dedicated coordinators that consist of an Obstetrics and Gynaecologist specialist (site leader) and nurses who are responsible for monitoring data entry. Data entry was performed by transferring antenatal records from a woman’s antenatal record book into the NOR database followed by entering/updating obstetric records during hospitalization as an outpatient, inpatient, or during delivery. These data are then centrally collated by a project managing team at the NOR headquarters office responsible for cleaning, analyzing, and publishing obstetric data.

Data from 2015 were utilized as the NOR undergoes data cleaning and quality control every two years before data publishing. At the point where this study was conducted, the latest cleaned and quality-checked data was from 2015. Besides that, based on the NOR annual reports from 2010 to 2020, the preterm birth rate for 2015, i.e., 12.4% (N = 151,268), was still well above the latest global prevalence of 10.6% estimated in 2014 (N = 139.95 million with an uncertainty interval of 9.0-12.0 million) [6,7]. Moreover, the preterm birth rate in Malaysia beyond 2015 was observed to decline. From 2017 onwards, the preterm birth rate dropped further to below the global prevalence, i.e., 11.7% (N = 142,946) in 2016, 8.9% (N = 152,615) in 2017, 7.0% (N = 63,917) in 2018, 6.7% (N = 67,960) in 2019, and 6.6% (N = 71,538) in 2020 [19,20].

Study population

The reference population refers to all births of women residing in Malaysia who attend government healthcare facilities. On the other hand, source population refers to all births registered with the NOR in 2015. Lastly, the selection of subjects refers to all live births (spontaneous) registered with the NOR in 2015 which fulfilled the inclusion and exclusion criteria of the study.

Study criteria

The inclusion criteria of the study were as follows: (1) all spontaneous live births (vaginal deliveries) of women who attended government healthcare facilities; (2) all spontaneous live births of women registered on the NOR database who delivered (only once) between January 1, 2015, to December 31, 2015; and (3) all spontaneous live births of women who received antenatal care at government healthcare facilities (booked cases).

The exclusion criteria of the study were, first, all spontaneous live births of women who were neither Malaysian citizens nor permanent residents. All foreigners and non-permanent residents were excluded as these individuals may have not received the same antenatal exposure, namely, antenatal care, compared to the local mothers selected in this study. Second, all spontaneous live births that were a result of multiple births (i.e., all non-singleton births) were excluded. Third, all spontaneous live births of women who conceived through assisted reproductive technology (i.e., all pregnancies as a result of non-spontaneous conception) were excluded. Live births resulting from twin pregnancies and assisted reproductive technology were excluded to reduce problems with multicollinearity and interaction effects. Highly correlated independent variables usually result in large standard errors for the estimated beta coefficients (or slopes) of these variables. Fourth, all spontaneous live births of women who did not have first-trimester dating scans (i.e., all late bookers) were excluded. This was done to ensure that only live births of pregnancy with accurate estimated delivery dates obtained through early dating scans were selected to confidently define the birth outcome (dependent variable) as preterm or term birth. Fifth, all spontaneous live births of women who were non-parous (i.e., all nulliparous women) were excluded. This was done because several antenatal risk factors under the evidence-based factor division of medical and pregnancy conditions, i.e., include a history of obstetric complication(s).

Sampling method

Universal sampling was performed as population data of all deliveries in 2015 was downloaded directly from the registry’s database as the source population (N = 151,268). Selection of subjects (N = 61,472) was based on the study inclusion and exclusion criteria. Subsequently, missing data management was performed which finally led to the study subjects (N = 49,416).

Sample size estimation

The sample size formula suggested by the MedCalc software online guide based on the number of events per variable was applied [21]. In this study, the number of covariates (final number of independent variables) involved (k) was 38. The value of (p1, p2), was obtained from the NOR 2017 annual report whereby out of 151,268 deliveries, 87.6% term births and 12.4% preterm births were recorded in 2015 [6]. Thus, p = min (0.876, 0.124) = 0.124. Hence, n = 10 (38)/0.124 ≈ 3,065, i.e., the minimum sample size required for the multivariable analysis in this study. As the final total number of study subjects (n = 49,416) was beyond the calculated minimum sample size of 3,065, valid study inferences can be made about the reference population of this study.

Statistical analysis

Before conducting inferential analysis using binary logistic regression, using SPSS software version 25 (IBM Corp., Armonk, NY, USA), data management was conducted resulting in a complete dataset of study subjects (N = 49,416) with 38 independent variables and one dependent variable. The regression analyses involved several stages, including the univariable analysis, multivariable analysis, checking for multicollinearity and interaction effects, assessing model adequacy using the Hosmer-Lemeshow goodness-of-fit test, overall correctly classified classification table, and area under the receiving operating characteristics (ROC) curve.

During the univariate analysis stage, the likelihood ratio chi-square of the contingency table and likelihood ratio of the logistic model were used to identify statistically significant variables for all 38 explanatory variables. Variables with a p-value <0.25 were then selected for the multivariable analysis [22].

Clinically important variables were assessed at the multivariate analysis stage. However, only variables with a p-value <0.25 were selected, regardless of their clinical importance. Subsequently, multiple regression using forward selection and backward elimination technique (stepwise approach using likelihood ratio statistics) was applied to automatically determine statistically significant explanatory variables.

Significant explanatory variables from the preliminary main effect model were checked for multicollinearity and interaction effects. Problems with interaction effects between variables were considered to not occur when the p-value for the interaction term was not significant (p >0.05).

Thus, the preliminary model was developed. Three model adequacy assessments were performed (Hosmer-Lemeshow goodness-of-fit test, classification table, area under the ROC curve). A classification table was presented and the ROC curve was plotted to determine how well the multiple logistic regression model discriminates the birth outcome.

The ROC curve plot is described as an alternative to the classification table [22]. The area under the curve (AUC), which ranges from 0.5 to 1.0, measures how well the model discriminates the birth outcomes. The larger the area, the greater the discrimination. The rule of thumb for AUC values is as follows: 0.5 indicates no discrimination to very poor discrimination, 0.6 indicates poor discrimination, 0.7 indicates acceptable discrimination, and, lastly, 0.8 and above indicates very good discrimination.

In addition, the ROC curve plots multiple coordinates of the sensitivity and 1 - specificity of the multiple logistic model based on different cut-points of the predicted probability [23,24]. Determining the optimal cut-point improve the prediction of the outcome on SPB, spontaneous term birth, or both outcomes together. To determine the optimal cut-point, a cut-point with the lowest index of union (IU) value was considered, where the formulation for the IU at cut-point c is as follows:

$IU(c)=|Se(c)-AUC|+|Sp(c)-AUC|$

where $Se(c)$ = sensitivity at cut-point c, $Sp(c)$ = specificity at cut-point c, and $AUC$ = area under the ROC curve [25].

## Results

In the multivariable analysis stage, multiple regression using the stepwise forward selection and backward elimination techniques (i.e., variable selection evaluated by likelihood ratio statistics) were used. Results from both regression techniques were compared. Of the 20 important explanatory variables (p < 0.25), both techniques selected 14 explanatory variables of the same variable name. These 14 statistically significant explanatory variables (p < 0.05) were syndromic condition, fetal sex, maternal height, ethnicity, history of preterm birth, diabetes mellitus, hypertension, antenatal color coding, intrauterine growth restriction, parity, BMI at booking, anemia at booking, age at pregnancy, and marital status. These explanatory variables (study factors) belonged to four of the six antenatal factor divisions, i.e., genetics and constitutional factors, medical and pregnancy conditions, nutritional factors, and sociodemographic factors. No explanatory variables were identified under the remaining two factor divisions, i.e., environmental toxicants and mental health, psychosocial, and behavioral factors. Table 1 summarizes the selected explanatory variables at the multivariable stage.

**Table 1:** Summary of the selected explanatory variables at the multivariable stage. Selected explanatory variables with p-value <0.05 at the multivariable stage (listed based on its factor divisions).

Factor division		Study factor
(a) Genetics and constitutional factors	i.	Syndromic condition
	ii.	Fetal sex
	iii.	Maternal height
	iv.	Ethnicity
(b) Medical and pregnancy conditions	v.	History of preterm birth
	vi.	Diabetes mellitus
	vii.	Hypertension
	viii.	Antenatal color coding
	ix.	Intrauterine growth restriction
	x.	Parity
(c) Nutritional factors	xi.	Anemia at booking
	xii.	Body mass index
(d) Sociodemographic factors	xiii.	Age at pregnancy
	xiv.	Marital status

Checking for multicollinearity and interaction effects

The model was then checked for multicollinearity and interaction effects. Multicollinearity analysis was not done as the logistic models involved only dummy variables. The interaction term with p-values shown in brackets was checked between the following nine pairs of explanatory variables that remained in the preliminary final model: antenatal color coding and age at pregnancy (p = 0.239); antenatal color coding and anemia at booking (p = 0.178); antenatal color coding and BMI at booking (p = 0.154); antenatal color coding and diabetes mellitus (p = 0.261); antenatal color coding and hypertension (p = 0.535); antenatal color coding and maternal height (p = 0.393); antenatal color coding and parity (p = 0.365); hypertension and intrauterine growth restriction (p = 0.911); and maternal age and syndromic condition (p = 1.000). There were no statistically significant interaction effects between the nine pairs of explanatory variables, as evidenced by their p-values of more than 0.05. At this point, a preliminary final model was achieved.

Assessing model adequacy

Before achieving the final model, three types of model adequacy assessment were performed: the Hosmer-Lemeshow goodness-of-fit test, the area under the ROC curve, and overall correctly classified (Classification table).

Hosmer-Lemeshow Goodness-of-Fit Test

The Hosmer-Lemeshow test goodness-of-fit test is shown in Table 2.

**Table 2:** Hosmer-Lemeshow goodness-of-fit test results. This test suggests that there is no significant difference (p = 0.678) between the observed and expected probability. Therefore, the test indicates that the model is fit, i.e., it is adequate to predict the birth outcomes. df = degree of freedom

Chi-square	df	P-value
5.729	8	0.678

Area Under the Receiving Operating Characteristics Curve

**Figure 3:**
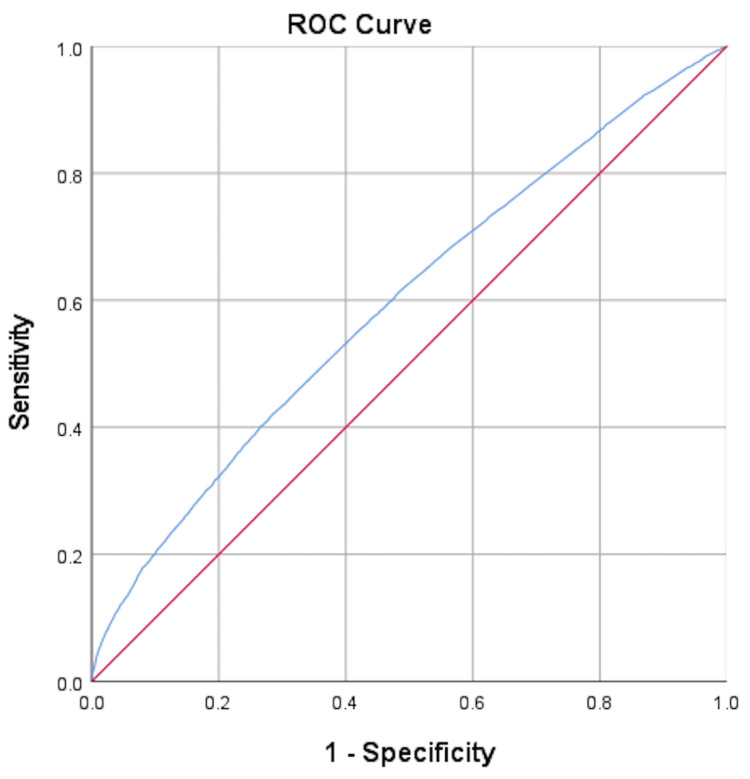
Area under the receiving operating characteristics (ROC) curve. The area under the ROC curve of the final model (AUC = 0.595, lower bound = 0.586, upper bound = 0.604, and standard error = 0.004).

The area under the ROC curve of the final model was plotted and is shown in Figure 3. The area under the ROC curve was 0.595 (lower bound = 0.586, upper bound = 0.604, and standard error = 0.004), with the value being close to 0.6. Based on the rule of thumb, the AUC value of 0.6 indicates poor discrimination for the birth outcome [22].

The ROC curve plots multiple coordinates (blue line) of the sensitivity (Y-axis) and 1 - specificity (X-axis) above the diagonal reference line (red line) of the multiple logistic model based on different cut-points of the predicted probability. In this study, the optimal cut-point that could improve the prediction of the birth outcome was calculated using the lowest IU value. The IU value was 0.058 calculated using the formulation for the IU at the cut-point [25].

The results showed (based on the calculated IU of 0.058) that the optimal cut-point for the predicted probability of the birth outcome was 0.094 with a sensitivity of 0.554 and a specificity of 0.578. Therefore, the results of the area under the ROC curve suggest that this model has limited use for the prediction of the birth outcome.

The Classification Table (Overall Correctly Classified Matrix)

Using the cut-point of 0.5 and formulae to measure model performance, the predictive accuracy was 0.905, the precision was 0.679, the sensitivity (true positive rate) was 0.004, and the specificity (true negative rate) was 1.000. Therefore, although the results of the classification table (cut-point 0.5) suggest that this model has an overall percentage of 90.5% in correctly classifying birth outcomes, with correctly classifying spontaneous term birth outcomes (100.0%), the model has poor performance in correctly classifying SPB outcomes (0.4%).

Subsequently, the classification table (model adequacy assessment analysis) was repeated using the optimal cut-point of 0.094 obtained based on the calculated IU of 0.058, as described in the Materials and Methods section on the area under the ROC curve.

Using the optimal cut-point of 0.094, the predictive accuracy was 0.575 and the precision was 0.121. The sensitivity (true positive rate) and specificity (true negative rate) were the same as calculated above, i.e., 0.554 and 0.578, respectively. Therefore, the results of the classification table using an optimal cut-point of 0.094 suggest that this model has an overall percentage of 57.6% in correctly classifying the birth outcomes, with correctly classifying spontaneous term birth outcomes at 57.8% and correctly classifying SPB outcomes at 55.4%.

The final model

Fourteen statistically significant explanatory variables remained in the final model (refer to Table 1). These associated antenatal factors of SPB among women attending government healthcare facilities in Malaysia in 2015 by multivariable analysis are shown in Table 3.

**Table 3:** Associated antenatal factors of spontaneous preterm birth among women attending government healthcare facilities in Malaysia in 2015 by multivariable analysis. Fourteen statistically significant explanatory variables with a p-value <0.05 remained in the final model. These associated antenatal factors of spontaneous preterm birth among women attending government healthcare facilities in Malaysia in 2015 by multivariable analysis are illustrated (presented in order of its factor divisions). OR = odds ratio; CI = confidence interval

Variable	Regression coefficient (β)	Standard error	Adjusted OR (95% CI)	Wald’s statistics	P-value
(a) Genetics and constitutional factors					
i. Syndromic condition					
None	-	-	1	-	-
Downs	1.683	0.622	5.382 (1.591, 18.213)	7.323	0.007
Edwards	2.335	1.431	10.329 (0.626, 170.495)	2.664	0.103
Patau	2.331	1.001	10.287 (1.445, 73.236)	5.417	0.02
Others	1.82	0.921	6.174 (1.015, 37.538)	3.907	0.048
ii. Fetal sex					
Female	-	-	1	-	-
Male	0.233	0.031	1.262 (1.187, 1.341)	55.84	<0.001
iii. Maternal height					
≥145 cm	-	-	1	-	-
<145 cm	0.332	0.099	1.393 (1.148, 1.692)	11.227	0.001
iv. Ethnicity					
Chinese	-	-	1	-	-
Malay	0.192	0.075	1.211 (1.046, 1.403)	6.523	0.011
Indian	0.346	0.106	1.412 (1.148, 1.738)	10.684	0.001
Others	0.296	0.085	1.345 (1.137, 1.590)	12.009	0.001
(b) Medical and pregnancy conditions					
v. Maternal history of preterm birth					
No	-	-	1	-	-
Yes	1.419	0.108	4.133 (3.344, 5.108)	172.56	<0.001
vi. Diabetes mellitus					
None	-	-	1	-	-
Gestational	0.092	0.065	1.096 (0.964, 1.246)	1.956	0.162
Pre-existing	1.127	0.356	3.087 (1.536, 6.206)	10.012	0.002
vii. Hypertension					
None	-	-	1	-	-
Chronic hypertension with superimposed pre-eclampsia	0.922	0.371	2.514 (1.215, 5.204)	6.171	0.013
Gestational	0.366	0.132	1.441 (1.114, 1.865)	7.726	0.005
Pre-existing	0.125	0.289	1.133 (0.643, 1.997)	0.187	0.665
viii. Antenatal color coding					
White	-	-	1	-	-
No code	0.846	0.176	2.331 (1.650, 3.293)	23.05	<0.001
Green	0.324	0.074	1.383 (1.195, 1.600)	19.01	<0.001
Yellow	0.673	0.085	1.96 (1.659, 2.316)	62.38	<0.001
Red	1.247	0.117	3.48 (2.766, 4.378)	113.36	<0.001
ix. Intrauterine growth restriction					
No	-	-	1	-	-
Yes	1.295	0.236	3.65 (2.299, 5.794)	30.14	<0.001
x. Parity					
1–4	-	-	1	-	-
≥5	0.152	0.056	1.164 (1.044, 1.298)	7.495	0.006
(c) Nutritional factors					
xi. Anemia at booking					
Normal (≥11)	-	-	1	-	-
Mild (9 to <11)	0.006	0.039	1.006 (0.931, 1.086)	0.02	0.888
Moderate (7 to <9)	0.168	0.058	1.183 (1.057, 1.325)	8.522	0.004
Severe (<7)	0.101	0.387	1.107 (0.518, 2.362)	0.069	0.793
xii. Body mass index at booking (kg/m^2^)					
Ideal (18.5 to <25)	-	-	1	-	-
Underweight (<18.5)	0.241	0.054	1.272 (1.145, 1.413)	20.15	<0.001
Overweight (25 to <30)	-0.151	0.038	0.86 (0.798, 0.927)	15.55	<0.001
Obese (≥30)	-0.097	0.014	0.908 (0.832, 0.990)	4.78	0.029
(d) Sociodemographic factors					
xiii. Age at pregnancy (years)					
18–35	-	-	1	-	-
<18	0.493	0.107	1.637 (1.327, 2.020)	21.13	<0.001
>35	0.064	0.054	1.066 (0.960, 1.184)	1.44	0.232
xiv. Marital status					
Married	-	-	1	-	-
Unmarried/Divorced/Widowed	0.334	0.104	1.397 (1.139, 1.714)	10.3	0.001

## Discussion

Prevention of SPB based on the four identified modifiable antenatal risk factors (presence of medical and pregnancy conditions, non-ideal BMI at booking, and anemia at booking) can be integrated into health service delivery packages, which exist in our health systems and involve links between maternal and child health services. These services fall on a continuum across the female reproductive lifecycle spanning from pre-pregnancy, pregnancy, birth, and newborn/postnatal up to the childhood stage.

The public health implications of the study findings can be described based on three levels of prevention. However, the discussion focuses on primary prevention, i.e., the pre-pregnancy phase. This encompasses interventions targeting women who may be conceiving in the near or distant future. The purpose is to reduce or eliminate causative risk factors (risk reduction) that, if left unaddressed, would result in poor pregnancy outcomes including preterm birth.

Presence of medical and pregnancy conditions

In this study, mothers with pre-existing diabetes had higher odds (adjusted odds ratio (aOR) = 3.09, 95% confidence interval (CI) = 1.54, 6.21, p = 0.002) of delivering prematurely compared to mothers without diabetes. Mothers with chronic hypertension with superimposed pre-eclampsia (aOR = 2.51, 95% CI = 1.22,5.20, p = 0.013) and gestational hypertension (aOR = 1.44, 95% CI = 1.11, 1.87, p = 0.005) had higher odds of experiencing preterm birth than mothers with no hypertension. Therefore, pre-pregnancy initiatives should prevent mothers from entering pregnancy with suboptimal glycemic and/or blood pressure control [26]. This includes raising community awareness, promoting early screening of risk factors and early referrals to pre-pregnancy care clinics.

In Malaysia, educational interventions are needed to enhance knowledge and awareness among women in the community. This is evident from a study that found an inverse relationship between knowledge of diabetes mellitus/hypertension in pregnancy and a pregnant woman’s glycemic/blood pressure level, including the risk of developing pre-eclampsia [27]. Another study reported that critical barriers to diabetes mellitus self-monitoring, for example, included a low perception of susceptibility to and severity of the illness, inadequate knowledge and skill regarding self-monitoring, and lack of motivation to perform self-monitoring [28].

Additionally, early detection of risk factors through health screening and treatment of type 2 diabetes mellitus and hypertension has shown a reduction in pregnancy complications and improved maternal survival and quality of life [29]. However, the health screening programs using questionnaires distributed at primary healthcare clinics in Malaysia among patients while waiting for their medical consultation appointments, such as the Borang Saringan Status Kesihatan (BSSK/W/1/2008), may not necessarily be effective. This may be due to the barriers in using the screening tool itself which was found to be less user-friendly causing reluctance among patients to voluntarily participate [30].

Ideally, women with pre-existing comorbidities should receive optimal control management before conception. This includes preconception counseling on contraception discontinuation recommendation based on desired glycemic and blood pressure control, as well as adjustment to non-teratogenic treatment type/modality in women on regular/life-long medication(s). However, due to resource barriers associated with the implementation of pre-pregnancy care, women with comorbidities are frequently managed at the antenatal care clinic instead (secondary preventive strategy) as they have entered pregnancy with uncontrolled comorbidities.

Intervention in the preconception period is perceived as “a window of opportunity” or an avenue to promote positive health behaviors because it is a time when women are more inclined to give up unhealthy lifestyles/habits [31]. Nevertheless, several ongoing issues were identified by Rahman et al. [32]. This includes the lack of awareness of the importance of preconception care among parents-to-be, particularly those from lower socioeconomic backgrounds. Additionally, lack of resources, specifically in organizational manpower and healthcare infrastructure constraints, has led to the “pre-pregnancy care clinics” occurring only as opportunistic sessions at primary healthcare clinics. Moreover, barriers arising from distorted cultural/religious perceptions of contraception/family planning as a medical recommendation/prescription to reduce the development of high-risk pregnancies are among the issues that need to be addressed. For example, reinforcing counseling by family medicine specialists at primary care settings on safe motherhood among certain groups of mothers, commonly religious extremists who tend to perceive the use of birth control to be an act of sin, i.e., ridiculing sustenance given by God.

Presence of non-ideal BMI (at booking)

In this study, underweight mothers had higher odds (aOR = 1.27, 95% CI = 1.15, 1.41, p < 0.001) of delivering prematurely than mothers with ideal BMI (18.5 to <25.0 kg/m^2^). Thus, promoting the aim of achieving an ideal BMI among to-be mothers during the pre-pregnancy phase is beneficial. A study by Ferrari and Siega-Riz in 2013 found that mothers who had an ideal BMI at their booking visit proceeded to have an appropriate weight gain than those who were not given such recommendations [33].

In contrast, women who enter pregnancy with a non-ideal BMI tend to experience inappropriate weight gain which puts them at risk of poorer pregnancy outcomes [34]. Although there is no clear global consensus on ideal weight gain, the guidelines by the Institute of Medicine in the United States recommend a range of allowed pregnancy weight gain based on pre-pregnancy BMI/booking BMI. According to this guide, healthy women with low weight (BMI: less than 18.5 kg/m^2^) should gain 12.5-18.0 kg, normal-weight women (BMI: 18.5-24.9 kg/m^2^) 11.5-16.0 kg, overweight women (BMI: 25.0-29.9 kg/m^2^) 11.0-14.0 kg, and obese women (BMI greater than 30.0 kg/m^2^) should only gain 5.0-9.0 kg during pregnancy [35].

In Malaysia, although pregnancy weight gain is monitored and managed accordingly throughout the antenatal care period, the crucial step of primarily preventing mothers from entering pregnancy with a non-ideal BMI is lacking. This could be due to the weakness of the existing pre-pregnancy clinic referral system. At some primary healthcare facilities, women who are referred to pre-pregnancy clinics are captured at their non-communicable disease clinic or antenatal/postpartum follow-up appointments. These women already have existing comorbidities and were likely to have a non-ideal BMI. Thus, a more effective referral system to the pre-pregnancy clinic should include capturing women from their earliest contact at the primary health clinic to initiate early nutritional and physical counseling.

For example, clinicians seeing women of childbearing age group at pre-marital health screen consultations and outpatient clinics should be instructed to ensure compulsory referrals to the pre-pregnancy clinic are made for these groups of women. Furthermore, the primary preventive strategy for women in the reproductive age group identified to be underweight should include screening for common causes of low BMI.

Generally, the management of underweight mothers focuses on nutritional counseling and monitoring of fetal growth to prevent adverse pregnancy outcomes, including intrauterine growth restriction, small for gestational age, and preterm birth. However, as discussed earlier, modifying dietary habits among local women may be challenging due to the larger social determinants influencing attitude, perception, and knowledge of a pre-pregnancy healthy diet. Therefore, targeted educational interventions are required to reach these special groups.

Presence of anemia (at booking)

In this study, women with moderate anemia had an increased risk of SPB (aOR = 1.18, 95% CI = 1.06, 1.33, p = 0.004). In other studies, SPB was associated with anemia, regardless of its severity. More importantly, anemia diagnosed early in pregnancy has been found to exert stronger associations, with adverse pregnancy outcomes than anemia diagnosed in later gestation [36]. Therefore, the focus should be on the prevention of anemia ideally before conception and during early pregnancy.

To prevent anemia, its cause should first be understood. The most common cause of anemia among women globally is iron deficiency which results from prolonged negative iron balance caused by inadequate dietary iron intake or absorption, increased iron losses because of menstruation, and increased needs for iron during pregnancy or growth periods [37]. In Malaysia, iron deficiency is responsible for 75-80% of all anemia cases among women in the reproductive age group. Young Malaysian women were found to have a low mean dietary iron intake, i.e., approximately only 10 mg/day, which is markedly below the recommended intake of 20-29 mg/day by the Malaysian health authorities [38]. Additionally, a study has reported that up to 75% of the main source of dietary iron among Malaysian women is from vegetable sources which has poor bioavailability, suggesting that only 5-10% is absorbed compared to the remaining 25% of animal dietary iron which has higher bioavailability [39].

Furthermore, 30-60% of menstruating women in Malaysia are victims of iron-deficiency anemia [40]. These women not only experience monthly background menstrual blood loss and thereby iron losses but may also have a past obstetric history of an average of two to three pregnancies and child deliveries, which contributes to further iron losses due to iron depletion during pregnancy (without supplementary iron) and iron losses due to bleeding at delivery [38]. Unfortunately, these women receive attention only when they become pregnant.

Therefore, in 2015, the World Health Organization (WHO) set one of the global nutrition community targets, i.e., to achieve a 50% reduction of anemia among women of reproductive age by 2025. This includes primary prevention of anemia through intermittent iron and folic acid supplementation (in menstruating women living in settings where the prevalence of anemia is 20%) and fortification of wheat and maize flours with iron, folic acid, and other micronutrients (in settings where these foods are major staples). The Centers for Disease Control and Prevention recommends that adolescent girls and women who do not require iron supplements be encouraged to eat iron-rich food and food that enhances iron absorption [41].

In Malaysia, preventive strategies are more focused on iron supplementation as a prophylaxis treatment of anemia among pregnant women, whereby the daily iron requirement of at least 27 mg is recommended from a dietary iron source with 25% bioavailability. However, the challenge would be in encouraging pre-pregnancy dietary changes emphasizing higher intake of animal-source iron as local studies have found that most women tend to resist recommendations to change dietary habits during the pre-pregnancy phase [38]. Furthermore, a recent study found that the prevalence of anemia among the Malaysian cohort participants was 13.8% (N = 102,388), with the majority having the microcytic and hypochromic type, implying iron deficiency as the main cause [42]. As the prevalence is below 20%, based on the WHO recommendation, the way forward for childbearing-age women in Malaysia should be to encourage iron-rich food. This further reinforces the need for comprehensive educational interventions to address resistance among women, particularly the socially vulnerable ones, to promote high-iron pre-pregnancy diets more effectively.

Although there are many other important causes of anemia worldwide, including helminth (intestinal worms) infestation, infections, and other micronutrient deficiencies, it is worth paying attention to genetic conditions/hemoglobinopathies. This is because thalassemia is one of the common autosomal recessive disorders which is highly prevalent in countries within the tropical belt including Malaysia [43]. Current estimation shows that 6.8% of Malaysians are thalassemia carriers who are likely to be affected with various degrees of anemia [44]. Nevertheless, both conditions of thalassemia minor and iron deficiency may co-exist within the same patient diagnosed with both a low hemoglobin and low serum ferritin level.

At present, primary prevention of thalassemia around the world follows a cascade of strategies that begins with creating awareness and enhancing knowledge regarding thalassemia. Through health education on thalassemia, the general public would be more motivated or receptive to screening, followed by the next level of prevention involving testing and genetic counseling for those who are carriers of the disorder.

Different screening approaches are used in different countries. In most countries, carrier screenings are being performed voluntarily, for example, based on different age groups or ethnic origin; obligatory basis, i.e., pre-marital screening; or family-centered (cascade) screening. In some Muslim countries, for example, the pre-marital screening programs for beta thalassemia are compulsory which aims at limiting marriage between carriers. In Cyprus, on the other hand, post-screening/testing decisions on proceeding with marriage and conception between carriers are left to the couple themselves. Therefore, due to the controversial criticisms of pre-marital screening, this approach has not been widely practiced across countries.

In Malaysia, the Ministry of Health established the Thalassaemia Prevention and Control Programme in 2004 to reduce morbidity and mortality related to thalassemia and the prevalence of blood transfusion-dependent thalassemia. Regarding primary prevention, the national screening program for thalassemia targets young school students between the ages of 16 to 17 years and first-degree relatives of known carriers of the beta thalassemia trait (family-centered/cascade screening).

Although such screening programs would have been preceded by health education, some local studies have reported the lack of awareness among certain individuals on the importance of screening itself resulting in reservations to screen. This is evident from a study that reported the mean thalassemia knowledge scores among parents to be unsatisfactory [45]. Parents with higher knowledge scores were associated with motivation to screen, i.e., permitting their child to be screened for thalassemia under the school’s national screening program. In addition, perceived stigma and discrimination attached to being a carrier of thalassemia were also identified as among the contributing factors for non-participation in the cascade screening. Thus, more attention needs to be given to step one of the primary prevention strategy which is to strengthen and enhance awareness and knowledge of thalassemia and its health consequences, as well as the economic and societal burden on individuals and families within communities.

Strengths and limitations

This study’s main strength lies in its novel approach within the subject area. Specifically, it is the first study to employ a big data methodology to explore antenatal factors of SPB across Malaysia. The NOR is a valid and reliable source of data, as it is the only database in Malaysia that collects hundreds of thousands of obstetric records through thoroughly trained data site providers ensuring its high quality. The NOR database’s standout feature is its ability to capture crucial data from women attending government healthcare facilities across Malaysia, covering the antenatal, intrapartum, delivery, and immediate postpartum stages. This not only provides accessible and affordable computerized data but also diverse and representative data of the study population. The statistical analysis used enhanced the study’s internal validity by controlling for confounding variables with logistic regression. Furthermore, the possibility of selection bias may have been obliterated as mothers with preterm births throughout Malaysia would have had an equal chance of being a subject in this study. Although this study does not encompass mothers who delivered in private settings, the NOR can be considered to represent the Malaysian population, as based on the Ministry of Health Malaysia annual report (2004), up to 80% of deliveries in Malaysia occur at government healthcare facilities.

This study had several limitations. First, some study factors were excluded due to unlisted variables in the NOR database, but proxy variables were used to overcome this. Second, misclassification bias from inaccurate diagnosis could have arisen during antenatal follow-up visits (possibly by junior medical officers at the primary care clinics) or at the delivery and/or postnatal care stage (possibly by junior medical officers at tertiary hospitals); however, this is addressed by the continuous monitoring by senior medical officers and specialists responsible for overseeing care. Third, recall bias could have occurred in mothers recalling their last normal menstrual period date to ascertain the estimated delivery due date. However, this is addressed with routine ultrasonography during the early trimester for gestational age assessment and confirmation. Fourth, measurement bias from the non-standardized instruments could have occurred, but this is addressed through quality control of medical measuring instruments including recalibration. Fifth, missing data was addressed through case deletion methods, which may have affected the overall model fitness. The Hosmer-Lemeshow goodness-of-fit test resulted in a non-significant p-value (p = 0.678), suggesting that the model is fit for predicting birth outcomes. The findings of this study have enhanced our understanding of SPB among women attending government healthcare facilities in Malaysia. Findings have revealed that there are modifiable risk factors of SPB allowing inferences to be made about the reference population of this study. However, due to the abovementioned study limitations, one must be cautious when generalizing the findings.

## Conclusions

Recommended measures to effectively reduce the rate of SPB in Malaysia include optimizing maternal biomarkers (glucose level, blood pressure, BMI, and hemoglobin level) by strengthening pre-pregnancy and antenatal care; empowering adolescents to aim for a healthy transition to adulthood (encompassing comprehensive sex education); proposing policies to support access to contraceptive services; and meeting family planning needs.
